# Molecular surveillance and temporal monitoring of malaria parasites in focal Vietnamese provinces

**DOI:** 10.1186/s12936-020-03561-6

**Published:** 2020-12-31

**Authors:** Bui Van Long, Genevieve Allen, Melanie Brauny, Le Thi Kieu Linh, Srinivas Reddy Pallerla, Tran Thi Thu Huyen, Hoang Van Tong, Nguyen Linh Toan, Do Quyet, Ho Anh Son, Thirumalaisamy P. Velavan

**Affiliations:** 1 Institute of Tropical Medicine, Universitätsklinikum Tübingen, Wilhelmstrasse 27, 72074 Tübingen, Germany; 2 Vietnamese-German Centre for Medical Research (VG-CARE), Hanoi, Vietnam; 3 Institute of Biomedicine and Pharmacy, Vietnam Military Medical University, Hanoi, Vietnam; 4 Department of Pathophysiology, Vietnam Military Medical University, Hanoi, Vietnam; 5 Vietnam Military Medical University, Hanoi, Vietnam

**Keywords:** Malaria, Species, msp1, msp2, Multiplicity of infection, Vietnam

## Abstract

**Background:**

While the World Health Organization (WHO) Southeast Asia region has the second highest incidence of malaria worldwide, malaria in Vietnam is focal to few provinces, where delayed parasite clearance to anti-malarial drugs is documented. This study aims to understand *Plasmodium* species distribution and the genetic diversity of *msp1* and *msp2* of parasite populations using molecular tools.

**Methods:**

A total of 222 clinical isolates from individuals with uncomplicated malaria were subjected to *Plasmodium* species identification by nested real-time PCR. 166 isolates positive for *Plasmodium falciparum* mono infections were further genotyped for *msp1* (MAD20, K1, and RO33), and *msp2* allelic families (3D7 and FC27). Amplicons were resolved through capillary electrophoresis in the QIAxcel Advanced system.

**Results:**

Mono-infections were high and with 75% *P. falciparum*, 14% *Plasmodium vivax* and 9% *P. falciparum/P. vivax* co-infections, with less than 1% *Plasmodium malariae* identified. For *msp1*, MAD20 was the most prevalent (99%), followed by K1 (46%) allelic family, with no sample testing positive for RO33 (0%). For *msp2*, 3D7 allelic family was predominant (97%), followed by FC27 (10%). The multiplicity of infection of *msp1* and *msp2* was 2.6 and 1.1, respectively, and the mean overall multiplicity of infection was 3.7, with the total number of alleles ranging from 1 to 7.

**Conclusions:**

Given the increasing importance of antimalarial drugs in the region, the genetic diversity of *P. falciparum msp1* and *msp2* should be regularly monitored with respect to treatment outcomes and/or efficacy studies in regions, where there are ongoing changes in the malaria epidemiology.

## Background

Malaria is still one of the overwhelming public health problems worldwide, and the World Health Organization (WHO) Southeast Asia region has the second highest incidence of malaria with a total of 7.9 million cases in 2018 [1]. Although there are encouraging reports of declined malaria morbidity and mortality in Vietnam since last two decades [2], malaria prevalence in central and southern provinces is still high, in particular provinces bordering Laos and Cambodia [3]. Although 40 out of 63 provinces in Vietnam are declared malaria-free, only a few individual provinces contribute to a third of the cases in the country each year [3], with an additional 1600 imported cases contributing to the burden, a phenomenon first reported in 2018 [1].

In Vietnam, *Plasmodium falciparum* (64%) and *Plasmodium vivax* (35%) are the most important malaria parasites. Artemisinin-based combination therapy (ACT), especially using dihydroartemisinin piperaquine (DHA-PPQ), is the first-line of treatment [1]. One of the main obstacles to malaria control is the parasites’ ability to develop artemisinin resistance [4], which is inherently defined as delayed parasite clearance [5, 6]. Resistance to artemisinin is well documented in Vietnam, Western Cambodia and Thai-Myanmar borders [7]. Since a significant proportion of the Vietnamese population lives in malaria endemic areas, where resistant phenotypes have been reported, studies to decipher the genetic diversity and multiplicity of infections (MOI) of *P. falciparum* are essential to understand the intensity of transmission, epidemiological patterns and virulence of the parasites and, in particular, to evaluate measures aimed at malaria control [8–11].

The diversity of *P. falciparum* is usually determined by the evaluation of the extent of the polymorphism of the merozoite surface proteins *msp1* and *msp2*, which are expressed on the surface of the merozoites during the erythrocytic stage of the life cycle of *P. falciparum* [12, 13]. The *msp1* gene on chromosome 9 consists of 17 different blocks, and block 2 is highly polymorphic and consists of three different allelic families: MAD20, K1 and RO33. The *msp2* gene on chromosome 2 contains highly polymorphic central repeats in block 3 and is distinguishable for two allelic families: 3D7 and FC27. Both *msp1* and *msp2* are highly immunogenic and are considered potential blood stage vaccine candidates [14, 15]. These two genetic markers are often used to indicate the number of parasitic strains present in a single host, which is useful in distinguishing between recrudescence and reinfection in drug efficacy studies [16]. The clonality of infection is the number of distinct clones/strain/genotypes per isolate (infected individual). More than one clone/genotype therefore implies polyclonality. Consequently, the mean number of genotypes or clones per infected individual (isolate) is defined to be the “multiplicity of infection” (MOI). A high MOI indicates that a single host carries multiple parasite strains and is associated with drug resistance of *P. falciparum* with excessive parasite transmission in holoendemic areas [16] .

In Vietnam, the predominant *msp1* and *msp2* alleles in 2012 [17] are MAD20 [18] and 3D7 [19], respectively. This study aims to determine the distribution of the *Plasmodium* species and subsequently the different *msp1*, *msp2* genotypes and to estimate the MOI in clinical isolates from malaria endemic areas in Vietnam.

## Methods

### Study area and sampling

Written informed consent was obtained from all study participants. The study was approved by the Institutional Review Board of Vietnam Military Medical University, Hanoi, Vietnam. The study included 222 clinical isolates collected from adult individuals with uncomplicated malaria (mean age = 29.1 ± 9.5; 88% female). An individual who presents with symptoms of malaria and a positive parasitological test (microscopy or RDT) but with no features of severe malaria is defined as having uncomplicated malaria. Clinical isolates were collected in Vietnamese provinces, which included Dak Lak, Gia Lai and Dak Nong between years 2017–2019. The three provinces (Dak Lak, Gia Lai and Dak Nong) are adjacent to each other and are part of the central highlands bordering Cambodia. Whole blood samples were collected from individuals and were microscopically confirmed for the presence of *Plasmodium* parasites. Blood samples were stored at − 20 °C until further use.

### *Plasmodium* species identification using nested real-time PCR

Genomic DNA was isolated from 50 µl whole blood using the QIAamp DNA Mini-Kit (Qiagen, Hilden, Germany) according to the manufacturer’s protocol. For detection and characterization of the *Plasmodium* species, a Taqman probe-based Pan-Plasmodium real-time PCR was used, as described earlier [20].

In short, the parasite DNA was amplified in a conventional PCR using the primers PLU5 and PLU6 [21]. The amplicons from the above PCR were used as templates in a single-plex nested real-time PCR assay for the differentiation of *Plasmodium* species. In each single-plex nested real-time PCR assay, distinct primers for *P. falciparum* [22], *P. vivax* [23], *Plasmodium malariae*, *Plasmodium ovale curtisi*, *Plasmodium ovale wallikeri* [20] are specifically used (Table 1). The assays were performed using SensiFAST™ Probe No-ROX Kit (Bioline, Tennessee, USA) in a LightCycler 480 Instrument II (Roche, Basel, Switzerland). Each clinical isolate was run in duplicates. The success of amplification is defined by respective Ct values that are smaller or equal to 40. All assays included a non-template control and positive control. The Ct values were calculated by default using the second derivative maximum method integrated in the LightCycler 480 software (version 1.5.1.62).

**Table 1:** List of primers used for plasmodium species identification using nested real-time PCR

Genus/species	Target	Primer ID	Primer sequence (5′ – 3′)	5′ modified	3′ modified	References
*Plasmodium*	18S rRNA gene	rPLU6-F	TTAAAATTGTTGCAGTTAAAACG			[21]
		rPLU5-R	CCTGTTGTTGCCTTAAACTTC			
*P. falciparum*	18S rRNA type S	PF-F	ATTGCTTTTGAGAGGTTTTGTTACTTT			[22]
		PF-R	GCTGTAGTATTCAAACACAATGAACTCAA			
		PF-Probe	CATAACAGACGGGTAGTCAT	HEX	MGBEQ	
*P. vivax*	18S rRNA type A	VIV-F	GCAACGCTTCTAGCTTAATCCAC			[23]
		VIV-R	CAAGCCGAAGCAAAGAAAGTCC			
		VIV-Probe	ACTTTGTGCGCATTTTGCTA	HEX	MGBEQ	
*P. malariae*	18S rRNA gene	PM-F	GGTGTTGGATGATAGAGTAA			[20]
		PM-R	CCCAAAGACTTTGATTTCTC			
		PM-Probe	AGGAAGCTATCTAAAAGAAACACTCAT	HEX	BHQ-1	
*P. ovale curtisi*	18S rRNA gene	POS-F	ATTTCAAAGAGTCATGGCGTTTCTG			[20]
		POS-R	TTGTAAAGGAGACACTTTCTTGAAATCG			
		POS-Probe	CTCCTTGGTCGATCTGCCCAGCACT	FAM	BHQ-1	[20]
*P. ovale wallikeri*	18S rRNA gene	POS-F	ATTTCAAAGAGTCATGGCGTTTCTG			
		POW-R	TGTAAAGGAGACAACTTTCTTGGAGCTA			
		POW-Probe	TTGATCGCCCAGCACTGACCATCT	HEX	BHQ-1	

HEX: 6-hexachlorofluorescein; FAM: 6-carboxyfluorescein; MGBEQ: minor groove binder eclipse quencher. BHQ-1: black hole quencher-1. rRNA: ribosomal ribonucleic acid

### *Plasmodium falciparum msp1* and *msp2* genotyping

The samples positive for *P. falciparum* were genotyped by nested PCR. The outer PCR was used to amplify conserved regions of *msp1* and *msp2* and then the nested PCR was used to amplify MAD20, K1 and RO33 allele families at the *msp1* gene locus and 3D7 and FC27 allele families at the *msp2* gene locus. The outer and inner PCRs were performed with published primers as described elsewhere [24].

In brief: for the outer PCR, the DNA fragment was amplified in a reaction mixture of 20 µl volume containing 1x PCR buffer (20 mM Tris-HCl pH 8.4, 50 mM KCl, 1.5 mM MgCl_2_), 200 µM dNTPs, 100 nM of each primer and 1U Taq DNA polymerase (Qiagen, Hilden, Germany) on an Eppendorf Nexus Gradient PCR Cycler (Eppendorf, Hamburg, Germany). The thermal cycle parameters for the first PCR amplification were: initial denaturation at 94 °C for 10 min, followed by 35 cycles of 30 s at 94 °C denaturation, 30 s at 55 °C annealing, 1 min at 72 °C extension, followed by a final extension of 10 min at 72 °C. The inner PCRs for the allele families MAD20, K1 and RO33 (*msp1*) and 3D7 and FC27 (*msp2*) were performed with the outer PCR templates of *msp1* and *msp2*, respectively. All five reactions were performed independently in 25 µl reaction mixture containing 3 µl from the outer PCR template, 1x PCR buffer, 1U Taq polymerase (Qiagen, Hilden, Germany), 200 µM dNTPs, 100 nM forward and reverse primers (Eurofins Genomic, Ebersberg, Germany). The PCR reaction conditions are identical to those of the outer PCR, except that the annealing temperature was 61 °C for the *msp1* allele families and 56 °C for the *msp2* allele families. For the *msp1* positive controls, *P. falciparum* DNA was isolated from the strains Dd2, NF54 and 7G8 for the alleles MAD20, K1 and RO33, respectively. For the *msp2* positive controls, DNA was isolated from *P. falciparum* strains Dd2 and NF54 for alleles 3D7 and FC27, respectively.

The QIAxcel Advanced system (Qiagen, Hilden, Germany) was used to resolve the amplicons by capillary electrophoresis. All nested PCR products were performed according to the AM420 protocol using the QX-DNA size marker 50–800 bp (Qiagen, Hilden, Germany) and the QX alignment marker 15 bp/1 kb (Qiagen, Hilden, Germany), except for the nested PCR products of the *msp1* allele family K1. The nested K1 PCR products were performed using the AM420 protocol with the QX DNA size marker 100 bp − 2.5 kb (Qiagen, Hilden, Germany) and the QX alignment marker 15 bp/3 kb (Qiagen, Hilden, Germany).

**Fig. 1:**
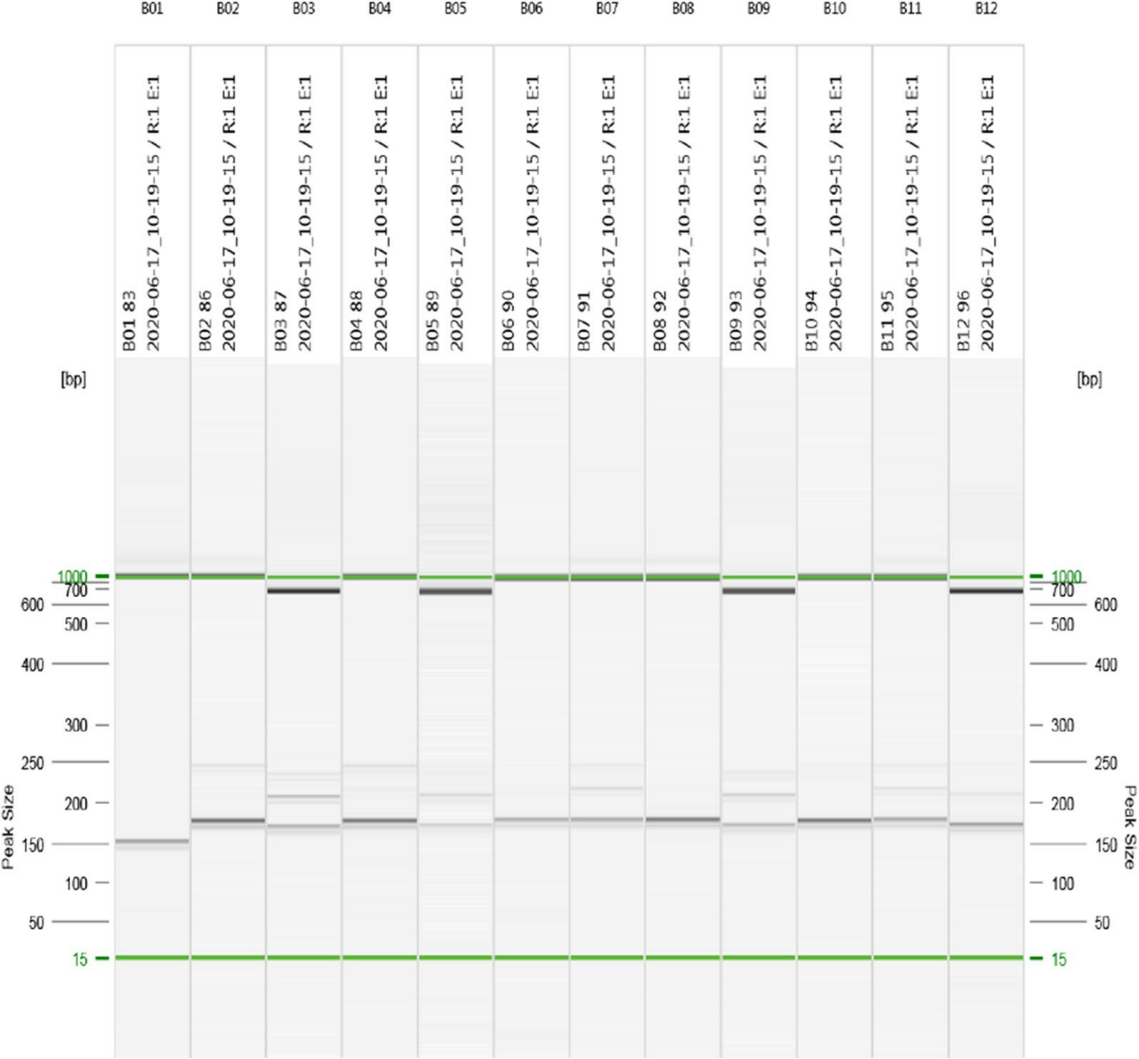
High-resolution capillary electrophoresis in a QIAxcel Advanced system, Amplicons were separated based on size and were subsequently analysed with QIAxcel ScreenGel software

The results were analysed with the QIAxcel Screen Gel software (Version 1.5.0.16, Qiagen, Hilden, Germany). All positive controls yielded a single peak, except the FC27 positive control, which clearly showed two peaks in each run (MAD20: 201, K1: 242 bp, RO33: 154 bp, 3D7: 237 bp, FC27: 469/623 bp). Each band represents one allele in the respective allele families (Fig. 1).

## Multiplicity of infection (MOI)

The MOI was defined as the mean number of *P. falciparum* genotypes per infected individual. The MOI was calculated as a proportion of the total number *of P. falciparum msp1* and *msp2* genotypes and the total number of PCR positive isolates. Isolates with only one allele at each locus were considered single infections. Infections with more than one allele at one or more loci were considered polyclonal infections.

## Results

### Plasmodium species identification

On screening 222 samples, 166 (75%) isolates were positive for *P. falciparum*, 32 (14%) isolates were positive for *P. vivax*, one isolate was positive for *P. malariae* and 20 (9%) isolates were positive for both *P. falciparum* and *P. vivax*, indicating co-infection. Furthermore, we did not find any isolates positive for *P. ovale curtisi* and *P. ovale wallikeri*. In total, 3 (2%) of the isolates were PCR negative.

### Genetic diversity and allelic frequency

All 166 isolates positive for *P. falciparum* by nested real-time PCR were subsequently genotyped for both *msp1* and *msp2* loci (Table 2). A total of 160 isolates were successfully amplified for *msp1* and 153 isolates for *msp2* loci, respectively. The MAD20 allelic family was predominant in 159 (99%) followed by K1 74 (46%) isolates, with the complete absence of RO33 allelic family. In *msp1*, 85 (53%) isolates were positive for MAD20 only and 73 (46%) isolates were positive for both MAD20 and K1, except one (0.6%) isolate that was positive for K1 only. In the *msp2* locus the 3D7 allele family was predominant in 148 (97%) and the FC27 allele family in 16 (10%) isolates. A total of 137 (90%) isolates were positive for 3D7 and 11 (7%) positive for both 3D7 and FC27. There were up to 7 different alleles for *msp1*, with allele sizes varying between 141 and 255 bp (MAD20) and 161–1270 bp (K1). There were up to 4 different alleles for *msp2*, with allele sizes varying between 248 and 650 bp (3D7) and 366–531 bp (FC27).

**Table 2:** Distribution of *msp1* and *msp2* alleles

Alleles	Positive N (%)
*msp1* (n = 160)	
MAD20 allelic family	159 (99%)
K1 allelic family	74 (46%)
RO33 allelic family	0 (0%)
MAD20 + K1 (polyclonal)	73 (46%)
MAD 20 (monoclonal)	85 (53%)
K1 (monoclonal)	1 (0.6%)
Mean MOI msp1 (± SD)	2.6 ± 1
*msp2* (n = 153)	
3D7 allelic family	148 (97%)
FC27 allelic family	16 (10%)
3D7 + FC27 (polyclonal)	11 (7%)
3D7 (monoclonal)	137 (90%)
FC27 (monoclonal)	5 (3%)
Mean MOI msp2 (± SD)	1.1 ± 0.5
Overall MOI (*msp1 + msp2*)	3.7 ± 1.2

### Multiplicity of infection (MOI)

Multiplicity of infection was calculated from the *msp1* and *msp2* genotyping results. A mean MOI of 2.6 ± 1 for *msp1* and a mean MOI of 1.1 ± 0.5 for *msp2*, and an overall MOI for *msp1* and *msp2* is 3.7 ± 1.2 observed. There is no significant distribution of MOI between age groups.

## Discussion

In this study, 222 clinical isolates were tested for *Plasmodium* species using sensitive real-time nested qPCR and were characterized the *P. falciparum* diversity, and MOI using molecular epidemiological tools.

From the nested real-time PCR results, 75% *P. falciparum* was observed, followed by 15% *P. vivax* and 8% *P. falciparum/P. vivax* co-infections. These results differ slightly from those of the WHO, where 64% was reported for *P. falciparum* and 35% for *P. vivax* in Vietnam [25]. These differences may be due to the fact that this study used a highly sensitive real-time PCR-based assay that is sensitive enough to detect submicroscopic infections. Another study showed that monitoring between December 2013 and January 2016 revealed 36% *P. falciparum*, 27% *P. vivax*, 25% co-infections with *P. falciparum* and *P. vivax* and 12% unidentified species from the same region [26]. In addition, it was reported that between 2006 and 2010, 70% of infections in Vietnam were due to *P. falciparum*. However, due to control measures, there has been a stronger impact on *P. falciparum* than on *P. vivax* since 2014, resulting in an almost equal ratio of both species [27]. Furthermore, co-infections with *P. knowlesi* and *P. vivax* have been documented in mosquitoes and humans in South Vietnam [28].

The use of ACT may have population-wide benefits in malaria control due to their effect in reducing the transfer of gametocytes, the sexual stage of the parasite, transmitted from humans to an *Anopheles* mosquito, during a blood meal [29, 30]. Rapid killing of the asexual parasite stages with artemisinin (99% daily killing rate) [29, 30] and, in combination with a partner drug, leads to a microscopically undetectable parasitaemia after 3 days of treatment [31]. Despite the decline of *P. falciparum* malaria in the last 10 years, and ACT being introduced as a component of comprehensive malaria control efforts, it is a challenge to reduce *P. falciparum* malaria cases in these provinces. The fact that the use of indoor spraying and insecticide-treated mosquito nets (ITN) is equally to contribute to these changes in the malaria epidemiology. Studies of the therapeutic efficacy of first-line treatment with DHA-PPQ at national sentinel sites have shown delayed parasite clearance in Gia Lai Province (2010), Dak Nong Province (2011), Quang Nam Province (2012), Khanh Hoa Province (2014) and Ninh Thuan Province (2015), with over 10% of patients being microscopically positive on day 3 after treatment initiation [32, 33].

Given the increasing importance of antimalarial drugs in the region, the genetic diversity of *P. falciparum msp1* and *msp2* should be regularly monitored with respect to treatment outcomes and/or efficacy studies in regions, where there are ongoing changes in the malaria epidemiology, as mentioned above [8, 9]. When magnified to understand the extent of parasite diversity in these provinces, the *msp1* MAD20 allelic family (99%) and the *msp2* 3D7 allelic family was predominant (97%). These results are in accordance with an earlier study from Vietnam [17]. Another longitudinal study in the region, in Myanmar between 2004 and 2006 and 2013–2015 shows changes in the *msp1* and *msp2* allele distribution over time [19]. For example, the RO33 prevalence changed from 0% (2004–2006) to 21% (2013–2015). The RO33 allele appears to occur in low frequency in this geographical region, as also in Myanmar where the presence of this type in parasites is also rare [34, 35]. Nevertheless, MAD20 and 3D7 remained predominant for almost two decades [19].

Studies with multiplicity of infections (MOI) are rare in Vietnam. The overall MOI of *msp1* and *msp2* in this study was 2.6 and 1.1, respectively, and these differ from those in Thailand and Laos, which are geographically closer to Vietnam. For *msp1*, the MOI in Thailand and Laos are 1.7 and 1.6, respectively, and for *msp2*, the MOI in Thailand is 2.5 [36, 37]. These differences may be due to differences in transmission intensity, population and geographical areas. The investigations were performed with a QIAxcel Advanced system, compared to previously reported investigations performed with conventional slab gel electrophoresis. This automated sensitive, high-resolution capillary electrophoresis performed DNA fragment analysis with the QIAxcel ScreenGel software with a power to discriminate alleles with a 3–5 bp resolution.

Against the background of the increasing importance of resistance to antimalarial drugs, which is reported in the region, a low to moderate genetic diversity was observed in the focal endemic provinces in Vietnam based on the genetic diversity of *P. falciparum msp1* and *msp2*. Routine monitoring of parasite genetic diversity has important implications for linking treatment outcomes and/or efficacy studies as the epidemiology of malaria changes.

## Data Availability

All related material and data are included in this manuscript.
